# Understanding Sleep-Wake Behavior in Late Chronotype Adolescents: The Role of Circadian Phase, Sleep Timing, and Sleep Propensity

**DOI:** 10.3389/fpsyt.2022.785079

**Published:** 2022-03-11

**Authors:** Christin Lang, Cele Richardson, Gorica Micic, Michael Gradisar

**Affiliations:** ^1^Department of Sport, Exercise and Health, University of Basel, Basel, Switzerland; ^2^College of Education, Psychology and Social Work, Flinders University, Adelaide, SA, Australia; ^3^Centre for Sleep Science, School of Psychological Science, University of Western Australia, Perth, WA, Australia; ^4^Adelaide Institute for Sleep Health, Flinders Health & Medical Research Institute, Flinders University, Adelaide, SA, Australia

**Keywords:** delayed sleep-wake phase, mood, dim-light melatonin onset, evening vigilance, depression, sleep deprivation, sedentary, school night

## Abstract

**Background:**

Adolescents with a late chronotype are at greater risk for mood disorders, risk-taking behaviors, school absenteeism, and lower academic achievement. As there are multiple causes for late chronotype, the field lacks studies on the relationship between mood, circadian phase, and phase angle of entrainment in late chronotype adolescents. Three objectives guide this explorative study: (1) to describe sleep, circadian phase, and phase angle of entrainment in late chronotype adolescents, (2) to explore how different levels of lateness are associated with sleep quality, sleep propensity, and mood, and (3) to investigate the influence of circadian phase on bedtime choice and sleep duration.

**Methods:**

Baseline data from 19 male adolescents (M = 16.4 ± 1.0 yrs), who were part of a larger intervention trial, were analyzed. Chronotype was measured with the Munich Chronotype Questionnaire, circadian timing via dim light melatonin onset (DLMO), and sleep habits with a 7-day sleep log. Further questionnaires assessed daytime sleepiness, sleep quality, and mood. Evening sleepiness and sustained attention were used as a proxy for evening sleep propensity.

**Results:**

On school nights, sleep duration averaged 7.78 h (±1.65), and 9.00 h (±1.42) on weekend nights. Mean DLMO was observed at 23.13 h (± 1.65), with a weekend phase angle of entrainment of 2.48 h. Regression fittings revealed a tendency for shorter phase angles with delayed DLMOs. Further analysis with chronotype subgroups revealed that this was only true for light and moderate late types, whereas extreme late types showed wide phase angles. Even though daytime sleepiness and sleep duration did not differ between subgroups, mood and sleep quality declined as lateness increased. Extreme late chronotypes experienced higher evening sleepiness, while slight late chronotypes showed higher evening attention. Chronotype but not DLMO predicted bedtime on school- and particularly weekend-nights.

**Conclusions:**

Our findings suggest that with increasing lateness, the likelihood of experiencing poor sleep quality and mood disorders increases. As DLMO did not predict bedtime, our data indicate that the factors contributing to a late chronotype are versatile and complex, particularly for extreme late types. Further studies involving a larger and gender-balanced sample are needed to confirm findings.

## Introduction

Individual differences in sleep-wake schedules can be classified as early, intermediate and late chronotypes. Early chronotypes (“morning larks”) rise early and feel their best in the morning, while late chronotypes (“night owls”) stay up late and feel most alert in the evening (1). The latter is well-known to many teenage parents. However, growing evidence supports a link between chronotype and increased risk for affective disorders (2–4). Specifically, a late chronotype has been linked to increased risk for depression, anxiety and substance abuse among adults (3). Few studies have investigated the relationship among adolescents. For example, adolescents with extreme late chronotype (delayed sleep wake phase disorder or DSWPD) show higher caffeine and alcohol consumption, and less sports participation (2). Considering that adolescence is a period of vulnerability for the onset of psychiatric diseases, which may be induced or exacerbated by insufficient and ill-timed sleep, this is of public health concern (5–8). Therefore, the current study aims to close this gap by investigating the specific role of different sleep and circadian related measures in late chronotype adolescents and their impact on mood.

Chronotype is closely related to the circadian rhythm (i.e., body clock), which regulates cyclical changes in cellular, molecular, and biological processes that repeat approximately once every 24 h. Natural daylight is the most potent timekeeper of the human circadian rhythm, hence artificial light exposure in the evening delays circadian timing thus sleep (9). Delayed circadian timing in adolescence is also driven by pubertal and developmental changes including a delay and lengthening of the circadian rhythm (10, 11). For example, the onset of melatonin secretion (a sleep-promoting hormone) and sleep timing becomes later with increasing age and pubertal status (12).

While delayed sleep seems to be a normal part of adolescent development, the misalignment between internal sleep-wake desire and early morning commitments (e.g., school) results in a reduced sleep opportunity. Attempts to initiate sleep at an earlier clock time may result in prolonged sleep onset times (4). The accumulated sleep debt during a school-week is then compensated on non-school days by sleeping in (13). This in turn leads to a reduced exposure to natural daylight in the morning which is necessary to reset and synchronize body clock timing, thus further delaying the circadian rhythm.

Morning bright light therapy with a gradual advanced sleep schedule and supplementation of evening melatonin are the recommended treatments for late chronotype adolescents (14). Although treatment responses are generally positive and remain the gold standard (15), a small but considerable proportion of adolescents show only small or negligible treatment effects on circadian markers, such as the timing of melatonin onset under dim light conditions (DLMO) (16). Moreover, not all observed phase-advances translate to earlier bedtimes, suggesting other factors in the etiology of late chronotypes (15). In fact, several studies have identified further factors that contribute to a late chronotype, such as homeostatic sleep pressure, circadian length, phase angle of entrainment, light exposure, and sensitivity (17–21). Circadian phase angle is the duration between circadian markers (i.e., DLMO) and sleep timing (i.e., onset or wake time) and often used as an indicator for circadian entrainment. Evaluations among adult populations revealed that only a subset of extreme chronotypes have primarily a circadian cause (17, 22–25). For example, in a recent study with patients diagnosed with DSWPD, only half presented with abnormally delayed circadian rhythms, while the other half showed abnormal phase angles between their biological rhythms and behavioral sleep-wake schedule (25). While both types are prone to suffer from negative physical and mental health consequences, the distinction may lead to a more tailored treatment approach. However, the literature lacks a concise definition toward “normal” and “abnormal” phase angles among adolescents. Among young and healthy adult populations, phase angles average 2–2.5 h (24, 26–29). Studies on extreme late chronotypes, including clinical samples with DSWPD, report similar (17, 30) or significantly shorter phase angles of 1.7 h (31). Shorter phase angles have been shown to be associated with a longer circadian rhythm (~25 h) and higher sleep need (20). However, little is known about these phase relationships in late chronotype adolescents. The phase angle of normal sleeping teens on free days has been reported in a study during summer holidays, with 1.18 h in the 9–12 year old cohort, and 1.65 h in the 13–16 year old cohort (32). For weekdays, a longitudinal study with a cohort of 15–16-year-old teens found phase angles closer to that of adults (2.05–2.17 h) (33). In contrast, only one study was found that reported the phase angle in late chronotype children (mean age 10 years), which averaged 1.22 h (34), and thus is larger than in an age matching cohort of healthy sleepers with 1.07 h (33).

In summary, late chronotype among adolescents has been associated with higher risk for mood disorders, risk taking behaviors, school absenteeism, and lower academic achievement. Current treatment approaches to phase advance circadian and sleep timing are widely accepted and promising, but moderate to large inter-individual differences highlight that not all adolescents benefit equally. Considering the multiple causes for late chronotype, and that adolescence is a critical developmental period for sleep, the field currently lacks data on the relationship between mood, circadian phase, and phase angle of entrainment among late chronotype school-aged adolescents.

Hence, the aim of this explorative study was three-fold: First, to describe differences in sleep, circadian phase, and phase angles of entrainment in late chronotype adolescents. Second, to explore how chronotype is associated with sleep quality, sleep propensity, and mood (i.e. depression, anxiety, and stress). Findings may highlight the importance of early interventions to improve sleep quantity and quality among this at-risk population. Thus, in order to design tailored interventions that address a delayed sleep period, and therefore sleep quantity and quality, our third aim is to explore the role of circadian phase on bedtime choice and sleep duration. Overall, we expect later bedtimes and thus shorter sleep durations on school nights, later circadian phase and reduced phase angle of entrainment, higher daytime sleepiness, lower evening sleep propensity, and worse mood for the more extreme late chronotypes compared to the intermediate chronotypes (5, 11, 35, 36).

## Methods

### Participants

The present explorative study investigated baseline data of participants that were part of a randomized controlled intervention trial, aiming to phase-advance circadian timing in late chronotype adolescents (trial registration DRKS00025322). The trial was approved by the Southern Adelaide Clinical Human Research Ethics Committee (SAC HREC application number OFR 100.16—HREC/16/SAC/90). Written informed consent was provided by all study participants and their parents or caregivers.

Inclusion criteria: male, aged between 15 and 18 years, sedentary (<60 min physical activity/week) (37), absence of evidence of sleep apnea and other pediatric sleep disorders (Paeditric Sleep Questionnaire; PSQ) (38), as well as psychological disorders, which have been linked to altered circadian rhythms [i.e., bipolar (39); ADHD (40), and Autism Spectrum Disorder (41)]. To meet inclusion criteria for a late chronotype, interested adolescents had to score >4.1 on the Munich Chronotype Questionnaire (MCTQ) (42). Exclusion criteria: travel across time zones in the 2 months prior to the study. Female adolescents were not recruited. The present data were drawn from an intervention trial with melatonin onset as the primary outcome. Due to conflicting evidence whether sex differences exist in circadian rhythms (43–46), we chose to include only male adolescents in the study. Further exclusion criteria involved intake of psycho-pharmaceuticals, as some antidepressants impact the biotransformation of melatonin (47, 48).

### Procedures

The study was advertised through social media. Interested participants contacted the first author of the study for an initial telephone screening interview. From the 62 adolescents that made contact, 28 male adolescents met inclusion criteria, and were invited with their primary caretaker to the Flinders University Sleep and Circadian Research Laboratory. Of these, 23 followed the invitation and consented to partaking in the study (Mage = 16.4 yrs, SD = 1.0). One week prior to their overnight stay, adolescents were instructed to complete a daily sleep log, while maintaining their habitual sleep-wake schedule during a regular school-week. Following 1 week of daily sleep log, dim light melatonin onset (DLMO) as a marker of circadian timing, was assessed via salivary sampling at the sleep laboratory. This ensured protocol compliance (e.g., continuous dim light condition, i.e., <10 lux) throughout the assessment, which started 4 h before and ended 2 h after participant's regular bedtime (max. 04:00 h). Study participants arrived at the sleep laboratory at 17:00 h. In the 72 h before and during saliva collection, adolescents were asked to avoid caffeine, nicotine, alcohol, and foods thought to impede habitual melatonin secretion (e.g., chocolate, bananas, tomatoes) (49). Self-reported daytime sleepiness and mood were assessed upon arrival (~17:30–18:00 h). Evening sleepiness and sustained attention were assessed in the last 3 h before their habitual bedtime. At other times, participants interacted with each other and staff members. Board/card games and a television were provided in the communal lounge room. Mobile phones were disallowed. Free access was provided to water. Meals and snacks were provided at set times consistently for all study participants, with no access to food at other times. Participants were monitored to ensure wakefulness until the completion of their last assessment (2 h after habitual bedtime). Thereafter, participants were allowed to sleep in and leave the laboratory (~11:00–13:00 h).

### Measures

#### Munich Chronotype Questionnaire (MCTQ)

The Munich Chronotype Questionnaire (MCTQ) was developed by Roenneberg et al. for ages 6 to 65 years (42). The self-rated questionnaire estimates chronotype based on the midpoint between average sleep onset and offset on school-free days (midsleep on free days: MSF), corrected for “oversleep” due to the sleep debt that individuals accumulate over a school week (MSFsc) (50). This proxy for chronotype is based on the assumption that sleep timing on school-free days is highly influenced by an individual's circadian clock. Therefore, chronotype (MSFsc) can only be calculated when participants can sleep in on their school-free days. Specifically, the MCTQ asks about bedtime, time spent in bed awake before deciding to turn off the lights, how long it takes to fall asleep, wake up time, and out of bed time for both school- and free days.

The MCTQ has been validated among various populations in Europe, Asia, North-, and South America and generally shows a high test-retest reliability (31, 51, 52). Validation against the gold standard for assessing circadian phase (DLMO) was high (53–56). Based on previously published population data, an MSFsc score of ≥4.1–5.0 was classified as slight late type, ≥5.1–6.0 as moderate late type, and ≥6.1 as extreme late type.

#### Dim Light Melatonin Onset (DLMO)

Salivary DLMO samples were taken half-hourly in dim light (<10 lux) using salivettes (Sarstedt, Newton, NC, USA). In line with the sampling protocol for adolescents developed by Crowley et al. (10), the measurements started 4 h before and finished 2 h after the participant‘s typical bedtime. Participants were seated for at least 5 min before and during each saliva sample, to minimize the masking effects of physical movement on endogenous melatonin production. Food and water consumption were only allowed after saliva collection to reduce contamination or dilution of the sample. Participants were instructed to gently chew on the cotton swab in their mouth and accumulate saliva for 2 min. Immediately after, samples were labeled and stored frozen at −20°C. For analysis, samples were thawed and centrifuged for 10 min at 2,500 rpm, the swabs removed from the casing, and the supernatant retained. A sensitive (4.3 pM) direct radioimmunoassay (RIA) using reagents from Buhlmann Laboratories AG (Allschwil, Switzerland) (57) was used to measure melatonin in the saliva. The intra-assay coefficient of variation (CV) was <10% at all times (mean = 4.5%). The inter-assay CV was 8.8% at 12.9 ppm and 13.1% at 104.5 ppm. The functional least detectable dose of the assay was 1.0 pg/ml. DLMO was calculated by linear interpolation across time-points when melatonin concentration increased to 4.0 pg mL or above (58).

#### Circadian Phase Angle of Entrainment

To determine circadian entrainment, the phase angle is calculated as the interval between circadian phase (i.e., DLMO) and sleep timing (i.e., onset or wake time). In the present study, phase angle of entrainment was calculated as DLMO-to-bedtime interval (DLMO_bedtime_). School-night bedtimes were calculated by averaging the bedtimes from Sunday to Thursday night. For weekend bedtimes, Friday to Saturday night was averaged.

#### Mood

Mood was measured with the short version of the Depression, Anxiety, and Stress Scale (DASS-21) (59). Each subscale consists of 7 items. Participants were asked to indicate how much each statement applied to them over the past week, using a 4-point Likert scale, ranging from 0 (“Did not apply to me at all”) to 3 (“Applied to me very much, or most of the time”) (60). The DASS-21 has been shown to be a valid and reliable measure of depression in adolescents, with adequate internal consistency (α = 0.76–0.90) (61), as well as satisfactory discriminant validity and convergent validity when compared to other measures of depression (62). This was confirmed in the present study (α = 0.76).

#### Evening Sleepiness and Sustained Attention as a Proxy of Sleep Propensity

The Karolinska Sleepiness Scale [KSS; (63)] was used to measure subjective sleepiness in the 3 h leading up to their averaged habitual bedtime. The KSS consists of a 9-point Likert-type scale, spanning 9 levels from 1 (extremely alert) to 9 (very sleepy, great effort keeping awake, fighting sleep). Adolescents were asked to circle the number that represents their current perceived level of sleepiness at 3, 2, 1, and 0 h before their averaged habitual bedtime.

Go/No-Go Task. Sustained evening attention was measured immediately after each KSS rating. The computerized Go/NoGo task (E-Prime v1.2, Psychology Software Tools, Inc., Pittsburgh, PA, USA, 2006) measures sustained attention in relation to inhibitory functions and consists of two visual stimuli presented in a random order. Adolescents pressed the space bar within 500 ms if the letter “M” was shown on the screen (Go stimuli). If the letter “W” was shown, they were instructed not to press any buttons (No-Go stimuli). A total of 80% of “M” (~200) letters were shown, in a quasi-random sequence wn across 8 min. Analyses were performed with commission error (falsely pressing the button in “No-Go” trials), as well as reaction time (RT) of correct Go-trials. The latter outcome has been used as a measure of sustained evening attention in previous research among adolescents and young adults (64, 65).

#### Daytime Sleepiness

To assess daytime sleepiness, the Pediatric Daytime Sleepiness Scale (PDSS), an 8-item self-report scale (e.g., “How often do you fall asleep or feel drowsy in class?”) was administered upon arrival at the laboratory (17:00 h) (66). Daytime sleepiness is a common symptom reported by adolescents with late chronotype (67–70). Responses to each item are measured on a 5-point Likert scale (e.g., 0 = “*Never*,” 4 = “*Always*”). Total scores range from 0 to 32, with higher scores indicating higher sleepiness. A total cut off score of 20 has been recommended for clinical samples (71). Drake et al. (66) reported good internal consistency (Cronbach α = 0.80); which was confirmed in the current study (Cronbach α = 0.80). The PDSS has been shown to be sensitive to chronobiological treatment and was used to measure changes in daytime functioning (72).

#### Sleep Diary

To assess subjective habitual sleep patterns, adolescents completed a daily sleep log at home over 7 consecutive nights before they came to the sleep laboratory. In the mornings, they indicated their bed- and rise-time, as well as perceived sleep onset latency (SOL), and number of awakenings during the night (WASO). Additionally, an 8-point Likert-type scale asked about sleep quality (1 = very bad sleep quality; 8 = very good sleep quality) each night. Nights were defined as weeknights if they went to school the next day; weekend nights were Friday and Saturday. To compute data, weekday and weekend sleep parameters were aggregated separately.

### Statistical Analysis

One-way ANOVA was used to compare baseline characteristics between groups (slight vs. moderate vs. extreme late chronotype). Repeated measure ANOVAs were conducted to compare the progression of pre-bedtime sleepiness and sustained evening attention (at 3, 2, 1, 0 h before bedtime) as a proxy for sleep propensity between chronotype groups. Test results with an alpha level <0.05 were reported as statistically significant. Due to the small sample size, effect sizes were considered when interpreting results (73). Effect sizes for ANOVAs [partial eta-squared (η^2^)] were regarded as small [S] if 0.01 > η^2^ < 0.059, medium [M] if 0.06 > η^2^ < 0.139, and large [L] if η^2^ ≥ 0.14 (74, 75). To test whether sleep duration is predicted by bedtime, wake-time, DLMO, or chronotype multiple linear regression models were conducted. Likewise, linear regression models were applied to test whether DLMO and chronotype predict sleep-wake times. All statistical analyses were performed using SPSS 28.0 (IBM Corporation, NY, USA).

## Results

### Participants

Four participants withdrew from the study before their overnight stay at the sleep laboratory (other commitments), while one decided to discontinue during the course of the data assessment (not willing to comply). Table 1 provides sleep and circadian characteristics for the 18 participants who completed the study (M = 16.44 years, SD = 1.04). Of these, 8 participants classified as slight late chronotype, 4 as moderate, and 6 as extreme late chronotype. The average BMI was 23.7, ranking 79.9 percentile in this age group (BMI percentile ≥5 and <85 = healthy weight). No significant differences between late chronotype subgroups (slight, moderate, extreme) were observed for age, *F*_(1, 6)_ = 0.78, *p* = 0.476, η = 0.09 and BMI, *F*_(1, 6)_ = 0.42, *p* = 0.959, η = 0.01.

**Table 1:** Descriptive statistics for sleep and circadian measures in chronotype subgroups.

**Characteristics**	**All (*****N*** **=** **18)**			**Slight late type (*****N*** **=** **8)**			**Moderate late type (*****N*** **=** **4)**			**Extreme late type (*****N*** **=** **6)**		
	**M ±SD**	**Min**	**Max**	**M ±SD**	**Min**	**Max**	**M ±SD**	**Min**	**Max**	**M ±SD**	**Min**	**Max**
Age in years	16.44 (1.04)	15	18	16.13 (1.13)	15	18	16.50 (1.29)	15	18	16.83 (0.75)	16	18
BMI	23.5 (5.1)	17.3	33.8	23.30 (4.83)	17.28	31.12	23.12 (6.33)	18.17	32.41	24.01 (5.68)	19.05	33.81
MCQ-score	5.56 (1.24)	4.25	8.27	4.53 (0.27)	4.25	5.00	5.27 (0.29)	5.13	5.70	7.13 (0.60)	6.64	8.27
TST (h)													
School night	7.78 (1.65)	5.28	11.83	7.55 (1.27)	5.67	9.75	7.55 (0.80)	6.40	8.25	8.23 (2.50)	5.28	11.83
Weekend night	9.00 (1.42)	5.70	10.72	9.56 (0.75)	8.50	10.50	8.96 (1.62)	7.00	10.42	8.29 (1.86)	5.70	10.72
Mid-sleep time (h:mm)													
School night (MSW)	3:53 (0:49)	2:38	5:55	3:46 (0:38)	2:50	4:52	3:46 (0:23)	3:12	4:07	4:06 (1:14)	2:38	5:55
Weekend night (MSF)	4:30 (0:42)	2:51	5:21	4:46 (0:22)	4:15	5:15	4:28 (0:55)	3:30	5:12	4:08 (0:55)	2:51	5:21
Bedtime													
School night	0.06 (1.09)	22.50	2.10	23.62 (0.97)	22.50	1.50	0.14 (1.61)	22.50	2.10	0.59 (0.74)	23.95	1.88
Weekend night	1.59 (1.67)	23.00	4.45	0.52 (1.18)	23.00	2.50	0.17 (1.06)	23.91	2.50	3.30 (1.15)	1.28	4.45
SOL (min)													
School night	19.11 (19.38)	1.80	72.00	14.68 (14.55)	1.80	36.25	23.60 (21.15)	5.40	54.00	22.02 (25.58)	5.00	72.00
Weekend night	21.36 (28.48)	4.00	120.00	21.91 (17.95)	6.00	52.00	41.31 (53.75)	4.00	120.00	7.33 (4.32)	4.00	15.00
WASO (min)													
School night	0.95 (2.59)	0	10.60	0.11 (0.18)	0	0.50	0.11 (0.13)	0	0.25	2.63 (4.20)	0	10.60
Weekend night	2.04 (4.36)	0	15.00	0.81 (0.92)	0	2.00	0	0	0	5.04 (6.84)	0	15.00
Wake-Up													
School morning	7.90 (1.59)	6.25	12.00	7.18 (0.87)	6.25	9.00	7.69 (0.94)	6.75	8.50	9.00 (2.15)	7.17	12.00
Weekend morning	10.49 (0.99)	9.50	12.50	9.93 (0.52)	9.50	11.00	10.16 (0.81)	9.50	11.15	11.45 (0.93)	10.25	12.50
Sleep quality													
School morning	5.59 (0.89)	3.40	7.00	5.49 (1.21)	3.40	7.00	6.07 (0.76)	5.20	6.60	5.48 (0.24)	5.20	5.80
Weekend morning	5.94 (1.30)	4.00	8.00	6.50 (1.12)	5.00	8.00	6.79 (0.25)	6.50	7.00	4.70 (1.10)	4.00	6.50
DLMO (h)	23.13 (1.65)	20.51	2.51	22.66 (0.96)	21.42	0.13	23.65 (1.70)	21.69	0.69	23.49 (2.36)	20.51	2.51
DLMO_bedtime_ (h)	2.48 (2.12)	−0.13	7.94	1.69 (1.40)	−0.13	4.08	1.57 (0.08)	0.67	2.22	3.98 (2.69)	0.49	7.94

*Data are presented as mean ± SD; MCQ, Munich chronotype questionnaire; TST, total sleep time; SOL, sleep onset latency; DLMO, dim light melatonin onset; WASO, wake after sleep-onset*.

### Sleep-Wake Timing, Sleep Duration, Sleep Quality, DLMO, and Phase Angle of Entrainment

Table 1 provides the descriptive statistics for sleep and circadian measures. The average sleep duration on school nights was 7.78 h (SD = 1.65), and 9.00 h (SD = 1.42) on weekend nights. Bedtimes on school nights ranged between 22.50–2.10 h and 23.0 h−4.45 on weekend nights. Large interindividual differences were also observed for wake-up times, which ranged between 6.25 and 12.0 h on school mornings, and 9.5–12.5 h on weekends. On average, adolescents had a weekend catch-up sleep of 1.22 h. Mean DLMO was observed at 23.13 h (SD = 1.65), with a weekend phase angle of entrainment for DLMO_bedtime_ of 2.48 h. Yet, the present sample revealed large interindividual differences on school-nights (M = 1.12; SD = 1.92; range = −2.56–5.36 h) and particularly on weekend-nights (M = 2.48; SD = 2.12; range = −0.13–7.94 h).

### Association Between Circadian Phase and Phase Angle of Entrainment

**Figure 1:**
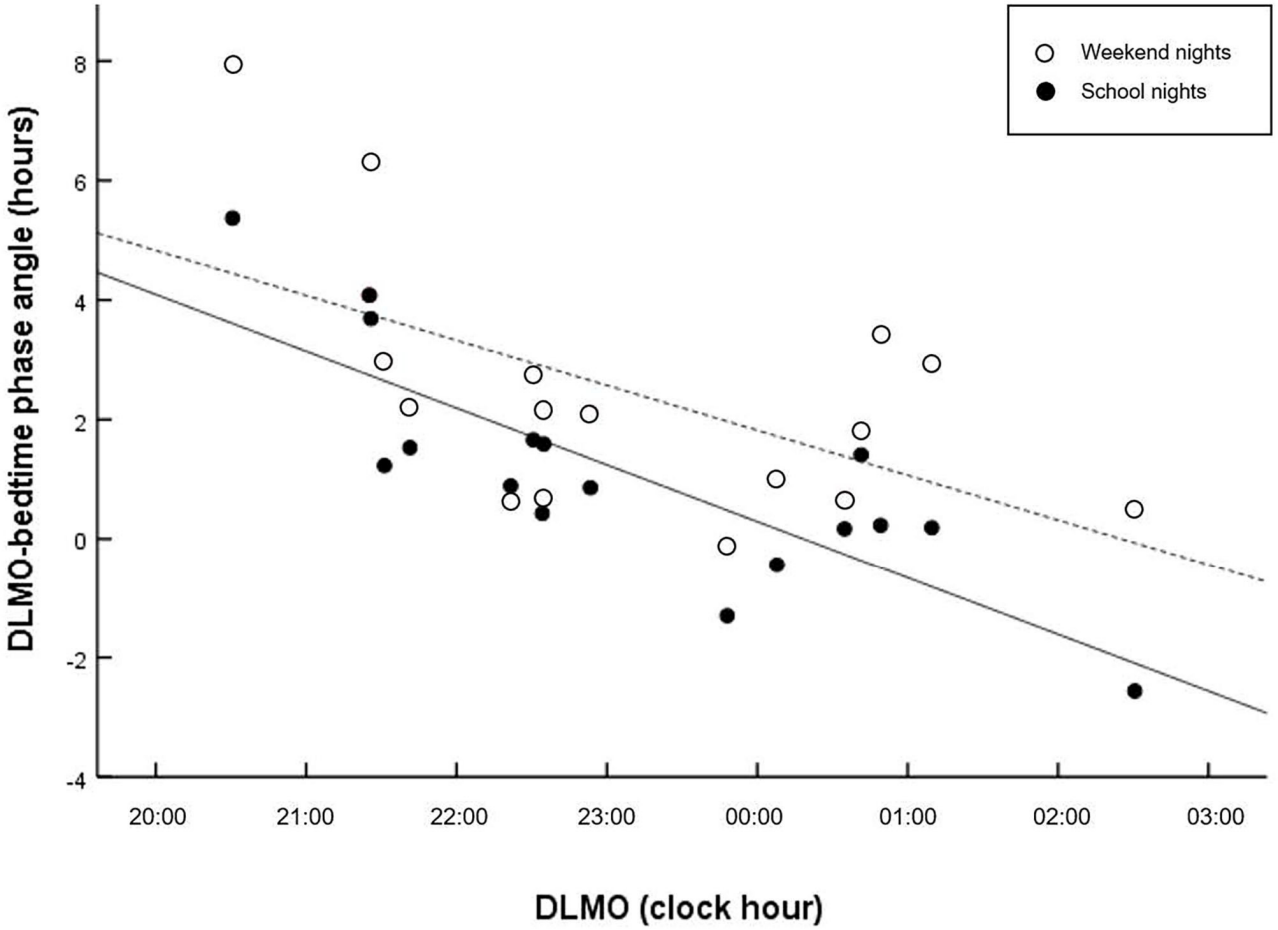
Scatter plots of phase angle relative to DLMO. Regressions are computed separately for school nights (solid line) and weekend nights (dashed lines). Shorter interval (phase angle) between circadian phase and bedtime is related to later circadian phase on school nights (*r* = −0.814, *p* < 0.001), and weekend nights (*r* = −0.583, *p* = 0.014).

**Figure 2:**
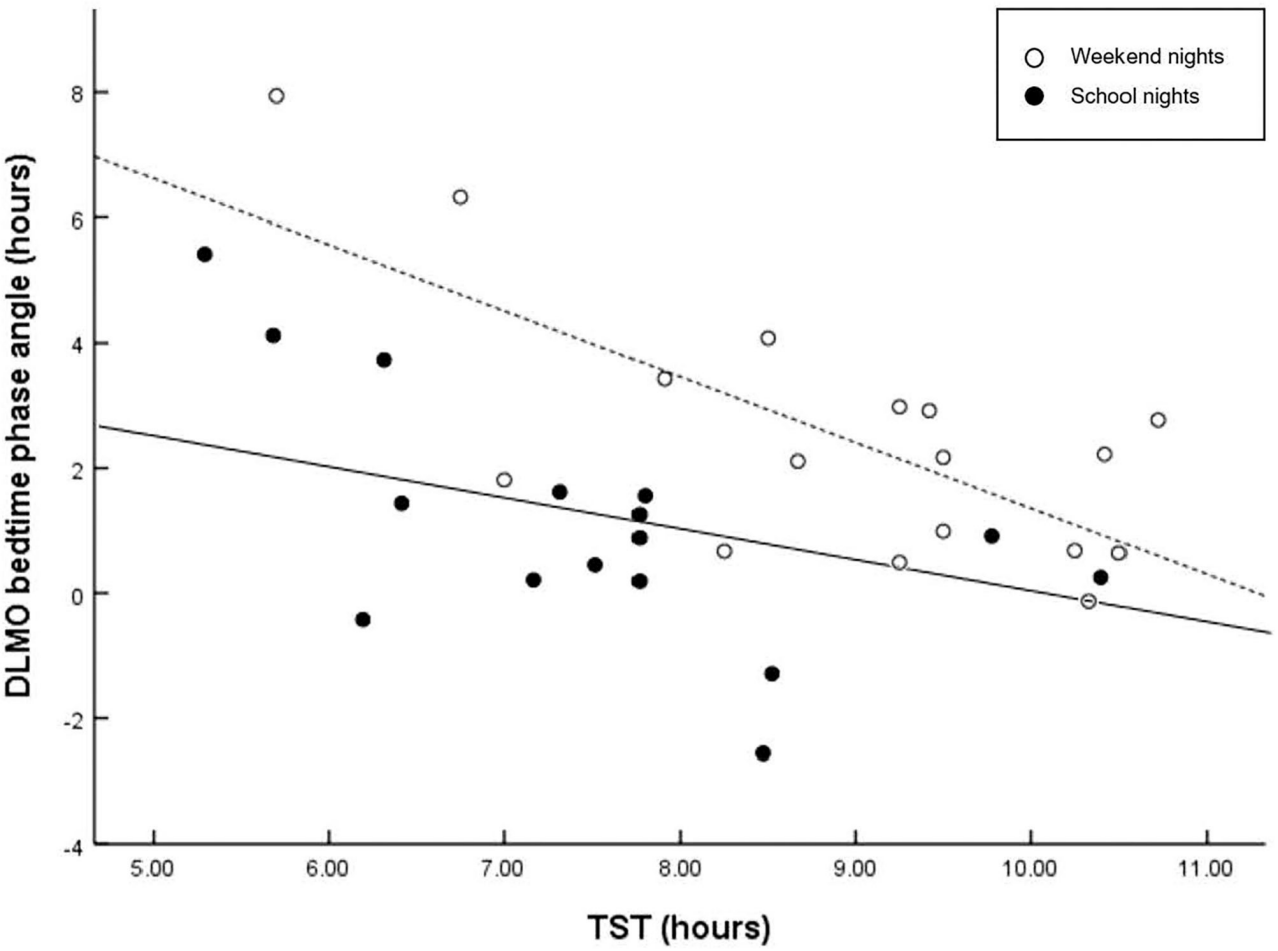
Scatter plots of phase angle relative to TST. Regressions are computed separately for school nights (solid line) and weekend nights (dashed line). Larger interval (phase angle) between circadian phase and bed time is related to longer sleep duration on weekend nights (*r* = −0.716, *p* = 0.001), but not on school nights (*r* = −0.436, *p* = 0.080). A wider phase angle of entrainment between DLMO phase and bedtime has been shown to be associated with a shorter intrinsic circadian period (tau), and reduced sleep need.

Figure 1 displays the correlations between the circadian phase marker DLMO and the respective phase angle of entrainment (DLMO_bedtime_) for school- and weekend nights. Regression fittings revealed that shorter phase angles were related to later circadian phases (DLMO). However, this relationship was less clear on weekend nights, *r* = −0.583, *p* = 0.014), with larger interindividual variations than on school nights, *r* = −0.814, *p* < 0.001), indicating that a late chronotype may be driven by factors other than circadian phase. In contrast, larger phase angles were related to longer sleep durations on weekend nights, but not on school nights (Figure 2).

### Differences in Sleep Patterns, Circadian Timing and Phase Angle of Entrainment in Slight, Moderate, and Extreme Late Chronotypes

Table 1 also provides the descriptive statistics for sleep and circadian measures for each chronotype subgroup (slight vs. moderate vs. extreme late chronotype). Inferential statistics for chronotype subgroups are represented in Table 2. No significant differences were reported for school nights (all >0.05). Yet, considering effects sizes due to the small sample size, morning wake-time and awakenings after sleep onset (WASO) were considerably later / more frequent among moderate and extreme late chronotypes, compared to their slight late peers. For weekends, significant differences were observed for bed- and morning wake-times, WASO, and perceived sleep quality. However, while large effect sizes for bed- and wake-up times are not of surprise (as chronotype is defined by these measures), sleep quality significantly decreased with increasing lateness in chronotype.

**Table 2:** Overview of inferential statistics for group (slight vs. moderate vs. extreme late type) (*N* = 18).

	**School days**			**Weekend days**		
	***F***	***p***	**η^2^**	***F***	***p***	**η^2^**
Sleep diary						
Bedtime	1.93	0.179	0.205	13.26	0.000	0.639
Wake-Time	2.79	0.093	0.271	7.76	0.005	0.508
SOL	0.36	0.707	0.045	1.89	0.185	0.201
WASO	2.16	0.150	0.223	2.59	0.010	0.256
TST	0.31	0.737	0.040	1.43	0.271	0.160
Sleep quality	0.50	0.618	0.066	6.69	0.013	0.549
Circadian timing and phase angle of entrainment						
DLMO	0.44	0.650	0.060	0.44	0.650	0.060
DLMO-Bedtime	0.11	0.896	0.016	2.87	0.091	0.290
Midsleep-Point	0.31	0.737	0.040	1.43	0.271	0.160

*Degrees of freedom: Always = 2, 16*.

**Figure 3:**
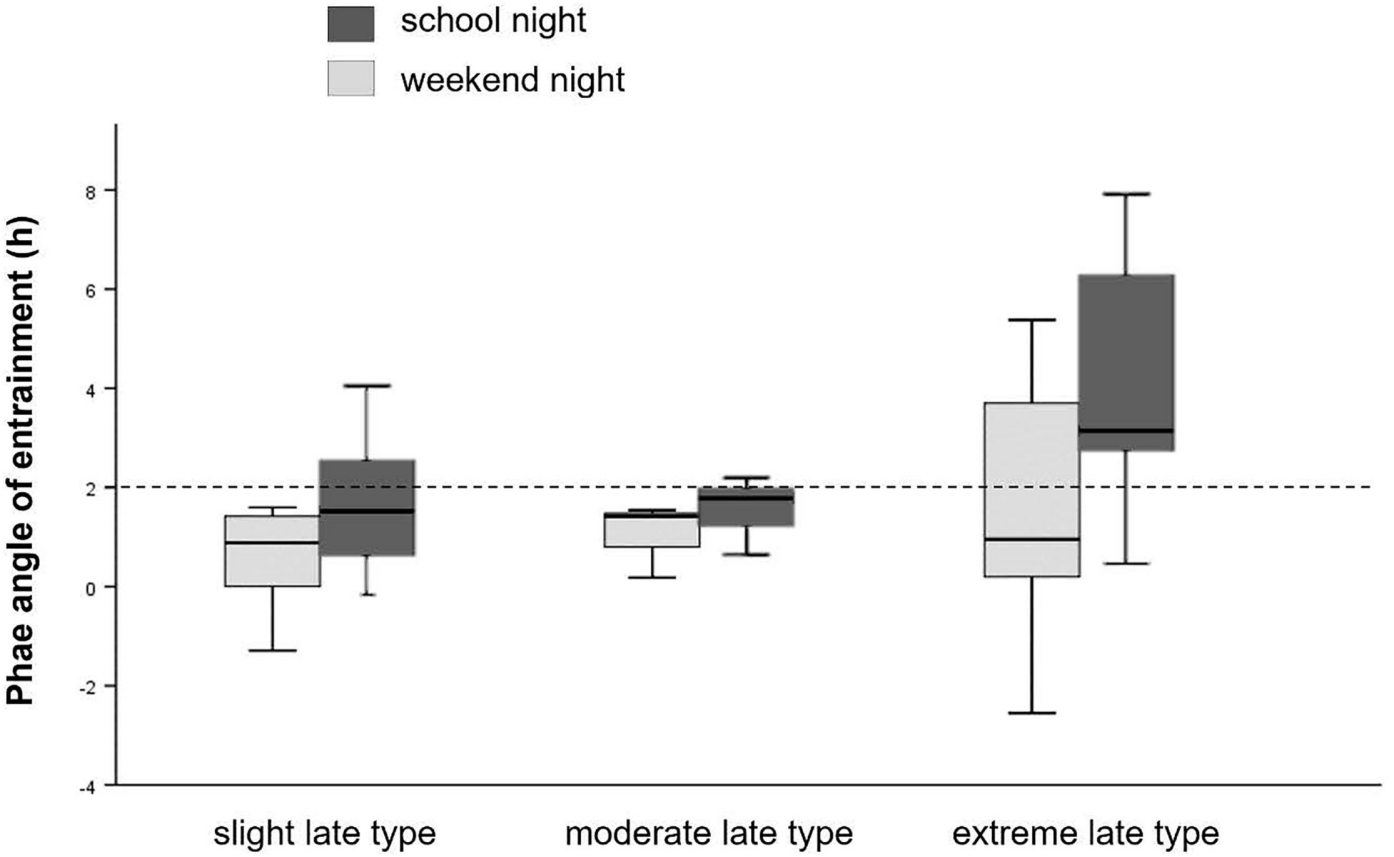
Phase angle of entrainment. Sleep onset timing in relation to DLMO for school- and weekend-nights, separately for chronotype. Dashed line represents the 2 h norm difference between DLMO and sleep onset timing.

As shown in Figure 3, phase angle of entrainment (DLMO_bedtime)_ was reduced among slight and moderate late chronotypes, but wide among extreme late chronotypes. The midpoint of sleep (MSF), defined as the clock time between sleep-onset and waking up, showed no significant differences among chronotype subgroups.

In summary, among this late chronotype sample, further analysis with chronotype subgroups revealed lower sleep quality and more variations in phase entrainment among extreme late types, regardless of MSF. Particularly, chronotype explained the variance in DLMO_bedtime_ on weekend nights (but not on school nights), in that large phase angles are more prevalent in extreme late chronotypes compared to slight and moderate late types, which tend to have reduced phase angles. Phase angles could not predict TST.

### Differences in Daytime Sleepiness and Sleep Propensity in Slight, Moderate, and Extreme Late Chronotypes

**Figure 4:**
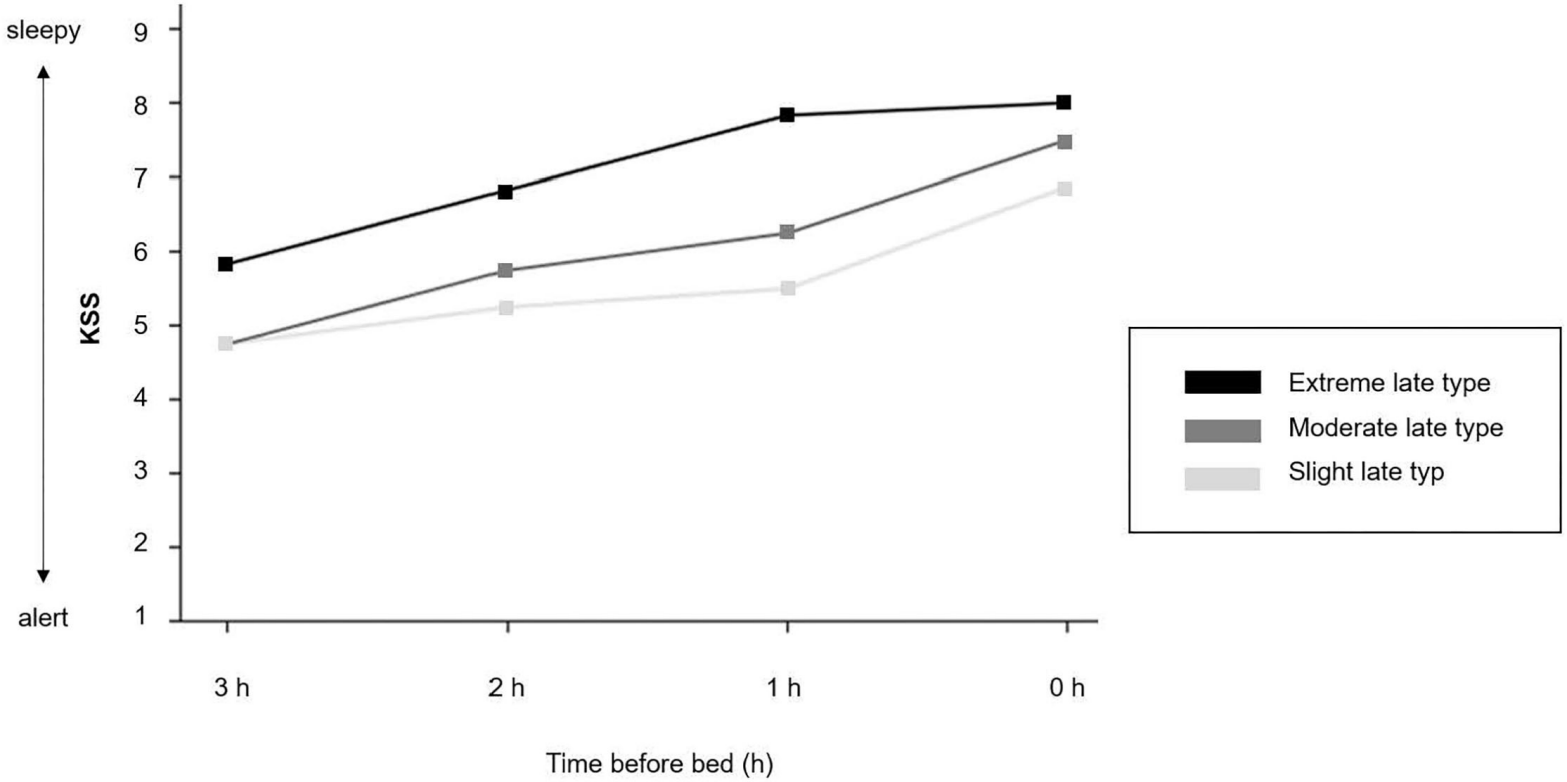
Evening sleepiness. Time course of the Karolinska Sleepiness Scale (KSS) (mean values). 1 = extremely alert; 5 = neither alert nor sleepy; 9 = very sleepy, great effort to keep awake.

Table 3 shows the descriptive and inferential statistics for evening sleepiness and sustained evening, attention separately for chronotype subgroups. The mean daytime sleepiness score was 15.5 (SD = 3.26), and did not differ between chronotype subgroups. As shown in Figure 4, evening sleepiness showed a steady increase among all three chronotypes from 3 hours before bedtime (M = 5.11, SD = 1.53) toward bedtime (M = 7.39, SD = 1.24). Yet, adolescents with an extreme late chronotype estimated their evening sleepiness significantly higher at all times, when compared to their moderate and slight late chronotype peers (slight and moderate vs. extreme late chronotype) *F*_(3, 17)_ = 7.95, *p* = 0.002, η =0.63. With regard to sustained evening attention, significant differences were found for commission errors, *F*_(3, 16)_ = 4.03, *p* = 0.01, η = 0.35, while reaction times remained constant, *F*_(3, 16)_ = 0.85, *p* = 0.488, η = 0.10.

**Table 3:** Descriptive and inferential statistics for daytime sleepiness, evening sleepiness, and sustained evening attention, as well as mood in chronotype subgroups.

**Characteristics**	**Slight late type (*****N*** **=** **8)**		**Moderate late type (*****N*** **=** **4)**		**Extreme late type (*****N*** **=** **6)**		**Statistics**		
	**M**	**SD**	**M**	**SD**	**M**	**SD**	**F**	***p***	**η^2^**
Daytime sleepiness	15.5	3.46	15.5	2.65	15.5	3.89	0.00	1.000	0.000
Evening sleepiness									
3 h before BT	4.75	1.83	4.75	1.26	5.83	1.17	1.00	0.390	0.118
2 h before BT	5.25	1.58	5.75	0.50	6.83	1.17	2.60	0.107	0.257
1 h before BT	5.50	1.41	6.25	1.50	7.83	0.75	6.00	0.012	0.444
0 h before BT	6.88	1.36	7.50	1.29	8.00	0.89	1.51	0.253	0.168
Evening attention									
RT 3 h before BT	309.63	44.23	319.75	35.82	317.83	26.51	0.13	0.879	0.017
RT 2 h before BT	304.25	47.48	336.25	22.59	323.50	42.13	0.87	0.440	0.104
RT 1 h before BT	310.38	43.13	356.25	43.87	326.00	35.12	1.69	0.219	0.184
RT 0 h before BT	353.75	41.31	353.75	41.31	327.83	44.97	0.731	0.498	0.089
Error % 3 h before BT	2.42	1.45	1.66	0.27	2.19	0.78	0.631	0.546	0.078
Error % 2 h before BT	1.45	0.30	1.94	0.21	1.99	0.50	4.56	0.028	0.378
Error % 1 h before BT	1.87	0.61	4.38	2.60	2.57	0.79	4.88	0.23	0.394
Error % 0 h before BT	1.68	0.63	2.79	0.57	2.17	0.90	3.21	0.069	0.300
Mood									
Depression	7.00	5.24	9.00	3.83	13.67	5.85	2.85	0.089	0.151
Anxiety	5.75	2.71	6.00	6.73	12.00	5.66	3.29	0.066	0.305
Stress	7.00	4.00	8.50	5.26	13.00	9.78	1.42	0.273	0.159

*M, mean; SD, standard deviation; BT, bedtime; RT, reaction time Go/NoGo task; error %, commission error percentage of Go/NoGo task; higher sleepiness scores reflect higher sleepiness, sustained evening attention, larger RT and error % reflect lower attention; Mood, higher scores represent higher prevalence of mood disturbances*.

In summary, while the level of daytime sleepiness was equally perceived among all three subgroups, subjective evening sleepiness was significantly higher among extreme late chronotypes, and sustained evening attention was significantly higher among slight late chronotypes, with less variance throughout the evening, when compared to their peers with a moderate and extreme level of lateness.

### Mood Differences in Slight, Moderate, and Extreme Late Chronotypes

**Figure 5:**
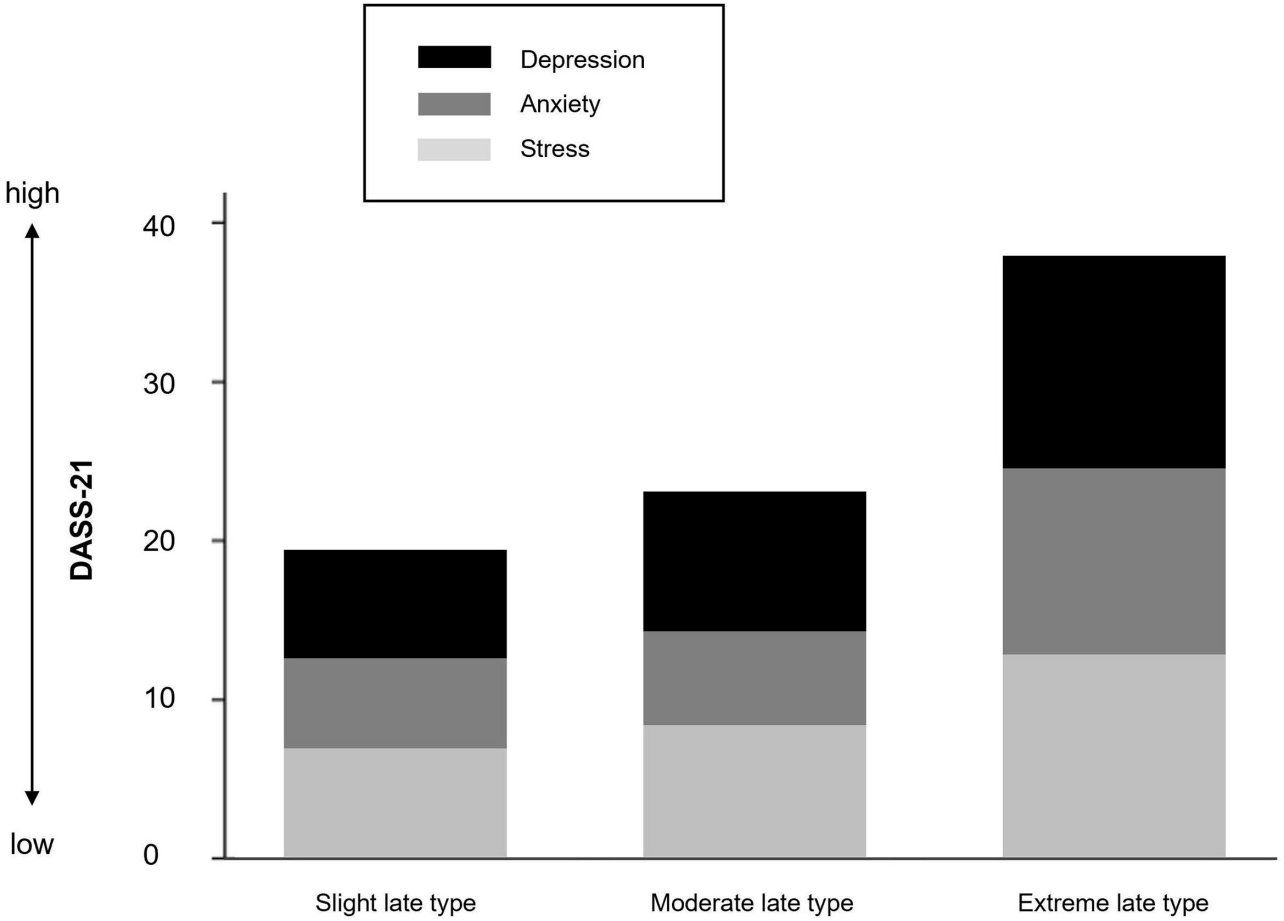
Stacked mean of depression, anxiety, and stress scores separately for chronotype.

Figure 5 shows the stacked mean of depression, anxiety, and stress scores separately for the three chronotype subgroups. Descriptive and inferential statistics are presented in Table 3. Even among this non-clinical late chronotype sample, a greater eveningness was associated with a higher risk tendency for depression *F*_(3, 16)_ = 2.85, *p* = 0.089, η = 0.15, and anxiety *F*_(3, 16)_ = 3.29, *p* = 0.066, η = 0.305, but not stress *F*_(3, 16)_ = 1.42, *p* = 0.273, η = 0.159.

### Predictors of Sleep Duration on School- and Weekend Nights

Table 4 shows the results of multiple linear regression models, representing the degree to which bedtime, wake-time, DLMO, and chronotype (MCTQ-score) can predict total sleep time (TST) on a school or weekend night in the present sample. TST on school nights was predicted by morning wake-time, β = 0.79, *t*_(16)_ = 4.69, *p* < 0.001, and not bedtime, DLMO, and chronotype. In contrast, TST on weekends was predicted by bedtime, β = −0.65, *t*_(17)_ = −4.66, *p* < 0.001, and chronotype β = −0.54, *t*_(17)_ = −2.11, *p* = 0.051. Again, DLMO had no predictive power on TST.

**Table 4:** Predictors of sleep duration on school and weekend nights.

	**TST school nights**			**TST weekend nights**		
	**β**	**SE**	***t***	**β**	**SE**	***t***
Bedtime	−0.54	0.35	−1.53	−0.65^***^	0.14	−4.66
Wake-time	0.79^***^	0.17	4.69	0.19	0.36	0.52
DLMO	0.26	0.26	1.00	0.11	0.23	0.49
MCQ	0.27	0.33	0.84	−0.54^*^	0.26	−2.11

*TST, total sleep time; bed- and wake times on school days were used as predictors of TST on school nights; bed- and wake times on weekends were used as predictors of TST on weekends;*

* p < 0.05, ^**^p < 0.01,

*** *p < 0.001*.

### Predictive Power of DLMO and Chronotype on Bed- and Wake-Up Times

Table 5 shows the outcomes of several multiple linear regression models, in which the predictive power of DLMO on bed- and wake-up time was weighed against the predictive power of chronotype. Overall, chronotype (MCTQ-score) but not DLMO predicted bedtime on school- and particularly weekend-nights. Similarly, wake-up times for both school- and weekend mornings were not influenced by DLMO, but chronotype.

**Table 5:** Multiple linear regression models on the predictability of DLMO and chronotype on sleep-wake times, separately for school- and weekend nights.

**Dependent variable**	**Predictor variable**	**β**	**SE**	***t***
School bedtime	DLMO	0.05	0.16	0.31
	Chronotype	0.39^+^	0.20	1.95
Weekend bedtime	DLMO	0.23	0.26	0.89
	Chronotype	1.08^***^	0.20	5.34
School wake-up	DLMO	0.28	0.24	1.14
	Chronotype	0.71^*^	0.27	2.69
Weekend wake-up	DLMO	0.24	0.15	1.66
	Chronotype	0.53^**^	0.15	3.5

+ *p < 0.07*

* *p < 0.05;*

** *p < 0.01;*

*** *p= 0.001*.

## Discussion

This is the first study to explore the relationship between sleep duration, bedtime and phase angle of entrainment among a non-clinical sample of late chronotype adolescents. In addition, we explored how the level of lateness impacts mood and sleep propensity. The use of subjective and objective measures was a particular strength of the study. This includes the gold standard for determining circadian phase—salivary DLMO in a light and temperature-controlled laboratory environment. A key finding was that even among this late chronotype sample, more extreme lateness was not associated with shorter sleep duration on school nights, but lower sleep quality and worse mood scores. While slight and moderate late types had reduced phase angle of entrainment, the opposite was true for extreme late types. Moreover, bedtime choice among extreme late chronotypes was not driven by circadian phase and sleep propensity, suggesting that other behavioral factors exert a stronger impact on the observed circadian misalignment.

Three aims were pursued within this explorative study design. First, we wanted to report on sleep, circadian phase, and phase angle of entrainment in late chronotype adolescents, followed by a comparison between three subgroups (slight vs. moderate vs. extreme late). In line with world-wide trends on adolescent sleep duration, the recommended amount of 9–9.25 h for optimal cognitive and emotional functioning among this age group (72, 76, 77) was not achieved during a regular school week with 7.8 h, but reached 9.0 h on weekends. Hence, the findings mirror sleep duration reports from age and region related populations (72). Interestingly, splitting the present sample into slight, moderate, and extreme late chronotypes, results show that weekend catch-up sleep was greatest among the slight late group (2 h), while sleep duration for extreme late chronotypes did not differ between school- and weekend nights. However, the latter group also showed the largest within-group differences in sleep duration, with both shortest and longest sleep periods among the entire sample (5.3–11.8 h on school nights and 5.7–10.7 on weekend nights). This leads to two assumptions: First, long sleep durations on school nights (with wake-up times far beyond school start) may indicate a severely delayed sleep phase and these individuals may have given up trying to adjust to a socially acceptable schedule. Second, some adolescents classifying as extreme late chronotypes are short sleepers. This notion is supported by the observed wide phase angle of entrainment of nearly 4 h, which has been shown to be associated with a shorter intrinsic circadian period (<24 h), and reduced sleep need (20, 78). However, this pattern of results is commonly observed among early chronotypes, and less among late chronotypes, at least when consulting data from adult samples (78). At first, this may seem contrary to our findings, particularly as our overall data show that a wider phase angle is associated with earlier circadian phase. Yet, for an adolescent with an early melatonin onset (~20:00), going to bed early may reduce the opportunity for social interaction, both digitally and in real life. Irrespectively of the sleep duration, a phase angle of more than 4 h suggests that sleep timing took place very late at an individual‘s intrinsic biological night. This misalignment between one‘s optimal sleep window (i.e., biological night) and actual sleep phase is known to impair restorative sleep and induce symptoms of insomnia.

For adolescents with a late chronotype, difficulty waking in the morning for school and daytime sleepiness are often the driving symptoms to seek care in sleep clinics. However, the physiological and psychological consequences are much broader than sleep-wake disturbance, as a growing body of research on circadian misalignment shows e.g., coronary heart disease, diabetes mellitus, mood vulnerability, and depression (79). These data highlight that non-circadian delayed sleep patterns are equally important to address than circadian induced late sleep-wake schedules. As for the present sample in general, shorter phase angles were seen in adolescents with later circadian phase, particularly on school nights. This phenomenon has previously been reported among late chronotypes, who aim to entrain to a socially accepted schedule. Despite their late circadian phase, these individuals are still aiming for sufficient sleep during a school week by selecting a bedtime much earlier in their biological night. However, it is not uncommon that this leads to chronic sleep onset problems, and the underlying circadian delay may only become apparent when presenting at a sleep clinic (34, 80). This assumption is supported with our findings, as phase angles were larger on weekends than on school nights. Moreover, the current state of research suggests that during puberty, phase angles increase with maturational stage. For example Lebourgeois et al. (81) showed that the relatively long sleep episodes and early bedtimes in toddlers leads to shorter phase angles (~0.67 h). Crowley et al. (10) measured phase angles at 1.18 h in a cohort of 9–12 year olds, and 1.65 h among the 13–16-year-old cohort of normal sleeping adolescents during summer holidays. In contrast, adults typically select a bedtime about 2 h after melatonin onset (23, 24, 26–28). In this respect, the phase angles between 1.69 and 1.57 h among our slight and moderate late sample indicates a normal entrainment for 16–18 year olds.

Nevertheless, the average circadian phase among our late chronotype sample was significantly later (23:08 h) than reports from age matching healthy sleeping cohorts on weekdays (~21 h) (33) and during summer holidays (21:30 h) (32). Interestingly, in a study on adolescents with evening preference by Dolsen and Harvey (82), which assessed affective mood in relation to DLMO, a comparable melatonin onset at 21:19 h was observed. Unfortunately, phase angles of entrainment have not been reported by authors, which would provide insights to what extend the sleep phase delay was induced by a circadian delay. In contrast, a recent study with high school students (14–18 years), who specifically report insufficient sleep on school nights (<7 h) and late bedtimes, DLMO averaged at 23:11 h (15), which is in line with the findings in our present sample. Only among an adolescent subgroup (12–16 years) with clinical diagnosed DSWPD, average DLMO occurred at 01:22 h (31). These heterogeneous findings are not unusual and support the notion of a “circadian” and “non-circadian” etiology of a late chronotype.

In line with this notion, our findings did not fully confirm our initial assumption that later chronotypes would present with a later circadian phase than their less severe peers. Specifically, comparing slight late with moderate late types (22.66 vs. 23.65 h) our assumption was partially confirmed. Yet, extreme late types did not show a more severe delay in circadian phase (23.65 h vs. 23.49 h) compared to moderate types. Thus, for extreme late chronotypes, in addition to the already existing circadian delay, further psychological and behavior-related factors presumably exacerbate the late sleep-wake pattern and observed circadian misalignment.

The second aim of the study was to explore how chronotype influences sleep quality, daytime sleepiness, evening sleep propensity, and mood. Generally, sleep quality decreased with increasing lateness. Yet, considering that a normal entrained circadian phase provides the best opportunity for restorative sleep (that is, an individual‘s sleep phase is in line with their biological night) our subgroup analyses appear to support that. Irrespectively of the circadian delay, our moderate late chronotypes perceived better and consistent sleep quality for both school- and weekend nights. This is in line with their phase angle, which not only corroborates the phase angles of a normal sleeping teenage cohort (32, 33), but also remains consistent between school- and weekend nights. Slight late types, on the other hand, slept at an earlier time in their biological night on school days, thus, perceived a lower sleep quality on those days. Yet, on weekends, when their phase angles were in line with those of the moderate late types, sleep quality equally improved. With regard to the extreme late chronotypes, which presented with a significantly larger circadian misalignment on weekends by staying up even later into their biological night, sleep quality was significantly worse than on school nights. Therefore, the general assumption that individuals with a delayed sleep-wake phase syndrome sleep just fine when allowed to choose their own bed- and rise time is not as simple. Based on our findings, the best opportunity for a good night‘s sleep is then achieved, when the bed- and wake time choice is also in line with one‘s intrinsic biological night. Unfortunately, individuals with extreme late chronotypes are struggling with just that, particularly those that present with a long circadian rhythm (i.e., 25 h instead of ~24 h) (21, 25).

Despite the different perceived sleep qualities upon awakening, no differences in daytime sleepiness were detected. However, the present sample compared extreme late types with slight late types, and such differences may only be detectable between early and late chronotypes. With regard to subjective sleepiness over the course of the evening, extreme late types rated their sleepiness significantly higher at all times compared to their earlier peers. Similarly, objectively assessed sustained evening attention was lower among moderate late and extreme late types. Therefore, our findings cannot confirm the hypothesis, that reduced sleepiness scores and higher evening attention in later chronotypes may be driving the delay of their sleep period. Moreover, higher evening attention among slight late types is in agreement with the observed shorter phase angles in this subgroup, implying that these adolescents have chosen a bedtime earlier in their biological night. Overall, while sleep propensity among our study sample might indeed be lower compared to normal sleeping adolescents, our findings highlight that it only explains to some extend the sleep-wake patterns of extreme late chronotypes compared to moderate and slight late types. In other words, genetics and bioregulatory changes of the circadian system during adolescent development play only one part in determining chronotype. The other chronotype-determining factors lay in the psychosocial behavior that inevitably leads to differences in “zeitgeber” signals, such as light exposure, food consumption, and physical activity patterns. A meta-analyses on the effects of electronic device use on adolescent's sleep revealed an increase in inadequate sleep quantity, as well as poor sleep quality, and excessive daytime sleepiness (83). Thus, psychosocial behaviors that strain sleep timing can perpetuate and aggravate a late chronotype.

As indicated in the introduction, late chronotype has repeatedly shown to increase the risk for mood disturbances in children and adolescents, particularly depression (4, 34, 82). Our findings corroborate this concerning fact, as symptoms of depression, anxiety, and stress were perceived considerably worse with increasing lateness. Yet, the strength of this effect between subgroups was somewhat surprising, given that only adolescents without a previous diagnosis of depression and anxiety were included in the study. Thus far, the association between late chronotype and mood disorders among adolescents is generally referred to inadequate sleep duration (84, 85). However, in the present sample, sleep duration did not differ between groups in that a shorter sleep duration would be responsible for the observed negative mood among extreme late types. In line with this, several studies have revealed that independently of sleep duration, later chronotype adolescents show increased symptoms of depression, and less positive mood compared to their early chronotype peers (86–88). Dolsen and Harvey (82) further investigated this assumption among 163 adolescents with an evening circadian preference. Higher negative affect was associated with a later DLMO. Referred to our sample, this would explain lower mood scores among the moderate and extreme late types compared to the slight late types. However, extreme late types did not present with later circadian phase than moderate late types, indicating that the additional circadian misalignment puts these youngsters at an even greater risk. Overall, the mechanism through which chronotype affects mental health is still poorly understood.

Our last aim was to explore the role of circadian timing on bedtime choice and sleep duration. As mentioned earlier, pervious research has shown that not all adolescents benefit equally from the recommended treatment to advance bed- and rise times. Morning bright light and exogenous evening melatonin are applied to phase advance circadian timing, and thus sleep phase (71). But to what extend does DLMO predict bedtimes among late chronotype adolescents? Among the present sample, DLMO did not predict bedtime and overall sleep duration, neither on school- nor on weekend days. This is in contrast to findings from a healthy normally-sleeping cohort of adolescents (32). Yet, our findings indicate that similar to patients with DSWPD (20), late chronotype adolescents show a greater variability in DLMO timing, as observed in the present sample. In contrast, sleep duration on school days was mainly predicted by wake-up time, whereas bedtime was the main predictor for sleep duration on weekends. Indeed, a large body of research supports the major role of early school start times on adolescent's sleep (89). As a result, schools that implement later morning schedules note increased sleep duration, improved class attendance, and reduced depressive symptoms (90).

Taken together, the findings of this explorative study show that as chronotype lateness increases, so does the risk for impaired mood and sleep quality. Moreover, extreme late types stood out not by an even later circadian delay, but by an additional misalignment between their intrinsic sleep timing and actual sleep phase. Considering that there are circadian and non-circadian reasons for a late chronotype, or as in case for the extreme late types a combination of both, studying circadian phase angles of entrainment among extreme chronotypes and psychiatric populations will further help to understand the link between chronotype and affective disorders.

## Limitations And Future Directions

We are aware that our work is not without limitations. First, the present study had a small sample size that limits the generalizability of the findings, particularly when dichotomized to subgroups. To this end, we supplemented inferential statistics with effect sizes that can be interpreted for their meaningfulness. However, it would be beneficial to replicate these explorative findings with a larger sample size. Second, not testing female adolescents could not provide reliable data regarding sex differences in mood and eating disorders in late chronotype adolescents (91). Third, ceiling floor effects among a sample composed only of late chronotypes may have prevented the detection of differences in sleep duration and daytime sleepiness. Future replication studies comparing early, intermediate, and late chronotypes may uncover such differences. In addition, a shorter time interval between the sleepiness ratings (e.g., 15 min instead of 60 min) might have allowed us to detect differences. Fourth, in our data, phase angle of entrainment was calculated using average bedtimes from a sleep log, which may have contributed to the low correlation between DLMO and bedtime. Fifth, a non-clinical sample was used for the study. In light of recent studies suggesting even higher prevalence rates of circadian rhythm disorders in psychiatric adolescents (92), further investigation of these issues with psychiatric samples may result in more clinically relevant findings. Last, we did not capture daylight exposure, which would shed further light on the etiology of adolescents late chronotype and their risk for lower sleep quality, and mood scores. Indeed, as stated in a recent Nature report, increasing daytime illuminance diminishes the impact of genetic factors that contribute to the interindividual differences in chronotype. In contrast, spending most of the day in relatively dim light conditions not only delays circadian timing, but also amplifies interindividual differences in circadian phase angle and preferred sleep-wake schedules (29). Thus, future studies in which daytime behavior and light exposure are compared between intermediate and late chronotype adolescents could shed further light on influenceable prevention and risk factors in support of a healthy sleep-wake schedule (i.e., active commuting to school, recess, outdoor sports, indoor light illumination).

## Conclusion

Taken together, our findings highlight that with increasing lateness, the risk to develop mood disorders increases. However, in line with a collective body of research on chronotype, our data indicate that the factors contributing to a late chronotype are versatile and complex. Thus, suggesting that there is no “one-fits all” treatment approach. Therefore, prevention measures early in adolescents' development should be considered. First and foremost, parents, teachers, and pediatricians, as well as adolescents should be educated that late sleep-wake rhythms in adolescence are more than just a normal, temporary condition. Misalignments, such as conflicts between one‘s internal body clock and outside social world (social jetlag), or between internal biological night and actual sleep phase obtained, lead to several negative physical and psychological health impairments through mechanisms that are yet to be fully understood. In light of this, our work provides insights into the sleep pattern, circadian phase, phase angle of entrainment, and sleep propensity of non-clinical late chronotype adolescents. Based on a recent finding that increasing daytime illuminance diminishes the biological risk factors to develop a late chronotype, we therefore recommend that schools evaluate indoor light conditions and time spent outdoors, so that adolescents are exposed to adequate light conditions during a school day.

## Data Availability Statement

The original contributions presented in the study are included in the article/supplementary material, further inquiries can be directed to the corresponding author/s.

## Ethics Statement

The studies involving human participants were reviewed and approved by Southern Adelaide Clinical Human Research Ethics Committee (SAC HREC application number OFR 100.16–HREC/16/SAC/90. Written informed consent to participate in this study was provided by the participants' legal guardian/next of kin.

## Author Contributions

CL and MG: conceptualization and methodology. CL and CR: data acquisition. CL: data pre-processing, data analysis, and writing of manuscript. CL, CR, GM, and MG: results interpretation. All authors revised, edited and contributed to the manuscript and approved the submitted version.

## Funding

This work was financially supported by the Swiss National Science Foundation (P2BSP1-165373) awarded to CL.

## Conflict of Interest

The authors declare that the research was conducted in the absence of any commercial or financial relationships that could be construed as a potential conflict of interest.

## Publisher's Note

All claims expressed in this article are solely those of the authors and do not necessarily represent those of their affiliated organizations, or those of the publisher, the editors and the reviewers. Any product that may be evaluated in this article, or claim that may be made by its manufacturer, is not guaranteed or endorsed by the publisher.
